# Scrub Typhus Presenting with Isolated Xerostomia

**DOI:** 10.4269/ajtmh.26-0003

**Published:** 2026-06-30

**Authors:** Soushi Nishiura, Masashi Narita, Yusuke Saishoji, Yasumori Izumi

**Affiliations:** ^1^ Department of General Internal Medicine, National Hospital Organization Nagasaki Medical Center, Omura, Japan;; ^2^ Division of Infectious Diseases, Department of Medicine, Okinawa Nanbu Medical Center and Children’s Medical Center, Haebaru, Japan;; ^3^ Department of General Medicine, Hakujyuji Hospital, Fukuoka, Japan

## Abstract

Scrub typhus is a mite-borne rickettsial infection that typically presents with fever, rash, and eschar; however, atypical manifestations may lead to delayed or missed diagnosis. The present report involves a case of a 65-year-old woman who presented with persistent xerostomia (dry mouth) as the sole ongoing symptom at the time of evaluation, without rash, eschar, or ongoing fever. She had a history of outdoor exposure in an endemic area, and laboratory testing revealed transient liver dysfunction and hyponatremia associated with polydipsia. Although empiric antimicrobial therapy was not initiated because of spontaneous clinical improvement, paired serological testing revealed a more than 8-fold rise in antibody titers against *Orientia tsutsugamushi*, confirming the diagnosis of scrub typhus. A review of recent cases without classical findings highlights frequent diagnostic delay and heterogeneous clinical outcomes. The present case underscores the limitations of relying on classical clinical hallmarks and emphasizes the importance of epidemiological context and clinical reasoning when evaluating atypical presentations of scrub typhus in endemic regions.

## INTRODUCTION

Scrub typhus is a mite-borne rickettsial disease caused by *Orientia tsutsugamushi* (*O. tsutsugamushi*) and transmitted to humans by larval trombiculid mites.^1^ It is endemic in Asia, particularly within the “Tsutsugamushi Triangle,”^2^ and its epidemiology is strongly influenced by geographic location, seasonality, and vector distribution, with increasing recognition of scrub typhus cases beyond traditionally endemic areas, likely reflecting previously unrecognized endemicity rather than true geographic spread.^3^^–^^6^ The clinical spectrum of scrub typhus is broad, ranging from mild illness to fatal disease. Although fever, rash, and eschar are considered characteristic findings, these manifestations are not universally present, and cases lacking typical features may be easily overlooked. Early diagnosis is essential because severe complications, such as acute respiratory distress syndrome, acute kidney injury, and meningitis, can be life-threatening.^7^^–^^9^ A case in which scrub typhus was suspected based on epidemiological exposure and clinical course despite the absence of rash and eschar is reported, and similar cases are reviewed to emphasize the diagnostic challenges of atypical presentations.

## CASE PRESENTATION

A 65-year-old Japanese woman presented with persistent xerostomia (dry mouth). Thirty days before admission, in March 2023, she had walked along a mountain trail while picking mandarins in Shimabara, Nagasaki Prefecture, where scrub typhus cases had been reported during the previous late fall to winter season. She reported no known exposure during that period. Fifteen days later, she developed a high fever of ∼39°C accompanied by fatigue and xerostomia. The fever and fatigue resolved spontaneously, but dry mouth persisted, and progressive polyuria prompted medical evaluation.

She had no significant past medical history, including scrub typhus, and was not taking medications. On admission, she was afebrile (maximum temperature 37.5°C), and her vital signs were stable. A physical examination revealed marked dryness of the oral mucosa, hepatosplenomegaly, and right inguinal lymphadenopathy. No rash or eschar was identified, including on the scalp, ear canals, axillae, umbilicus, or external genitalia.

Laboratory testing revealed mild anemia and liver dysfunction: a white blood cell count of 4,500/*µ*L (atypical lymphocytes 405/*µ*L), a hemoglobin level of 10.3 g/dL, a platelet count of 1.58 × 10^5^/*µ*L, an aspartate aminotransferase level of 305 IU/L, an alanine aminotransferase level of 288 IU/L, an lactate dehydrogenase level of 499 IU/L, and a C-reactive protein level of 0.28 mg/dL. Her serum sodium level was mildly decreased (133 mEq/L), with normal renal function. Viral hepatitis serology results were negative. Epstein–Barr virus testing indicated past infection. Antinuclear antibody test results were weakly positive (×40), anti–Sjögren syndrome-related antigen A antibodies were elevated, and anti–Sjögren syndrome-related antigen B antibody test results were negative; however, criteria for Sjögren’s syndrome were not met. Urinalysis revealed polyuria with low urine osmolality (181 mOsm/kg H_2_O). Computed tomography revealed hepatosplenomegaly and right inguinal lymphadenopathy.

Despite the absence of a rash or eschar, scrub typhus was considered because of recent outdoor exposure in an endemic area. Indirect immunofluorescence assays for *O. tsutsugamushi* and *Rickettsia japonica* were performed on admission by the Nagasaki Prefectural Institute for Environmental Research and Public Health. Empiric antimicrobial therapy was withheld because the patient remained clinically stable and exhibited spontaneous improvement, and close outpatient follow-up was ensured.

Marked polyuria (3,952 mL/day) with low urine osmolality and mild hyponatremia suggested excessive water intake. Fluid restriction reduced urine output and normalized urine osmolality, consistent with polydipsia-induced polyuria. The patient improved without complications and was discharged on hospital day 5.

Follow-up serological testing revealed a more than 8-fold rise in paired antibody titers against the Hirano/Kuroki strain of *O. tsutsugamushi*, confirming scrub typhus (Table 1). Molecular testing could not be performed because pretreatment blood samples were unavailable. No recurrence was observed, and liver enzyme and serum sodium levels normalized during follow-up.

**Table 1:** Paired IgM and IgG antibody titers against *Orientia tsutsugamushi* (six serotypes), *Rickettsia japonica*, and severe fever with thrombocytopenia syndrome virus

Antigen	Serotype	Antibody	Day 15 of Illness	Convalescent (day 34)
*Orientia tsutsugamushi*	Karp	IgM	80	320
		IgG	640	640
	Kato	IgM	40	80
		IgG	320	160
	Gilliam	IgM	160	80
		IgG	320	160
	Hirano/Kuroki	IgM	160	>1,280
		IgG	>1,280	>1,280
	Irie/Kawasaki	IgM	160	80
		IgG	320	160
	Shimokoshi	Testing not performed		
*Rickettsia japonica*	YH strain^10^	Below the detection limit		
SFTS virus		Negative		

SFTS = severe fever with thrombocytopenia syndrome. Paired IgM and IgG antibody titers against *Orientia tsutsugamushi* (six serotypes), *Rickettsia japonica*, and severe fever with thrombocytopenia syndrome virus. Antibody titers were measured using an indirect immunofluorescence assay on day 15 of illness and during convalescence (day 34). Values represent reciprocal titers. All assays were performed at the Nagasaki Prefectural Institute for Environmental Research and Public Health.

## DISCUSSION

The present case represents a clinically instructive atypical presentation of scrub typhus confirmed by paired serological testing, in which the patient lacked classical clinical features at presentation, including rash and eschar, and had no ongoing fever. According to the proposed disease spectrum of scrub typhus, this presentation corresponds to Category 4 (“easily overlooked”), characterized by the absence of fever, rash, and eschar.^11^ The diagnosis was supported by a history of outdoor exposure in an endemic region during the season when *Leptotrombidium scutellare*–mediated scrub typhus is prevalent. Xerostomia, the only persistent symptom in this case, has rarely been reported in association with scrub typhus. Although a direct causal relationship could not be confirmed because of the lack of salivary gland scintigraphy data, previous studies have suggested that *O. tsutsugamushi* may affect salivary gland function.^12^^,^^13^ The normalization of serum sodium and urine osmolality after water restriction supports primary polydipsia driven by xerostomia as the main mechanism of hyponatremia. However, given previous reports of syndrome of inappropriate antidiuretic hormone secretion in scrub typhus, a contributory role cannot be entirely excluded.^14^

The absence of eschar formation, often described as *scrub typhus without eschar*, is not fully understood but is likely multifactorial. Experimental studies involving nonhuman primate models have suggested that host immune responses, particularly cell-mediated immunity, may reduce bacterial burden and alter local skin manifestations.^15^ In addition, host factors such as underlying comorbidities and partial immunity from previous exposure—as suggested by the elevated acute-phase Hirano/Kuroki IgG levels observed in the present case (Table 1)—may attenuate local inflammatory responses, reflecting previous exposure or asymptomatic infection, and contribute to the lack of eschar formation. Variations in bacterial load, inoculation site, and strain-specific pathogenicity may also play a role.

To contextualize this atypical presentation, recent adult cases of scrub typhus without rash or eschar reported between 2019 and 2024 were reviewed (Table 2).^11^^,^^16^^–^^29^ Most patients presented with fever and nonspecific systemic symptoms, whereas the present case was unique in that xerostomia was the only persistent manifestation and fever had resolved by the time of presentation. Several cases experienced diagnostic delay because of atypical clinical features or concurrent infections, highlighting the limited diagnostic value of clinical presentation alone in the absence of classical signs.^24^^,^^26^

**Table 2:** Reported adult cases of scrub typhus without rash or eschar, 2019–2024

Ref.	Age/Sex	Nation	Clinical Presentation (excluding fever)	Diagnose Method	Accuracy	Pd (days)	Hd (days)	Outcome
11	62/M	Japan	Diarrhea	IFA	Probable	6	None	Recovered
16	33/M	India	Chills, AMS	ELISA	Possible	12	N/A	Recovered
16	76/M	India	Chills, AMS	ELISA	Possible	15	N/A	Recovered
16	22/F	India	Jaundice, stomachache	ELISA	Possible	14	N/A	Recovered
16	30/F	India	Jaundice, stomachache, itching	ELISA	Possible	8	N/A	Recovered
16	60/F	India	SOB, vomiting, AMS	ELISA	Possible	10	N/A	Death
16	39/F	India	Jaundice	ELISA	Possible	N/A	N/A	Recovered
16	30/F	India	AMS	ELISA	Possible	N/A	N/A	Recovered
16	45/M	India	AMS	ELISA	Possible	N/A	N/A	Recovered
16	45/F	India	AMS	ELISA	Possible	N/A	N/A	Recovered
17	26/F	India	Headache, chills, dizziness, vomiting	ELISA	Possible	4	N/A	Recovered
18	51/F	China	N/A	mNGS, PCR	Probable	7	3	Recovered
19	45/M	Nepal	Stomachache, dysuria	RFA	Possible	3	4	Recovered
20	21/M	India	SOB, AMS	ELISA, RFA, IFA, PCR	Probable	10	N/A	Death
20	30/F	India	Vomiting, stomachache	ELISA, RFA, IFA, PCR	Probable	11	N/A	Death
20	24/M	India	Headache, myalgia, body swelling	ELISA, RFA, IFA, PCR	Probable	8	N/A	Death
21	80s/F	China	Diarrhea, melena	DNA analysis	Probable	3	N/A	Recovered
21	70s/F	China	Chest tightness	DNA analysis	Probable	5	N/A	Recovered
22	38/F	India	Chills, headache, hearing loss	Serology	Possible	15	N/A	Recovered
23	35/M	China	N/A	Metacap	Probable	10	N/A	Recovered
24	23/M	India	Headache, cough, SOB, myalgia	ELISA	Possible	7	N/A	Recovered
24	28/F	India	Cough, myalgia	ELISA	Possible	N/A	N/A	Recovered
25	52/F	China	Chills, headache, myalgia, dysuria	mNGS	Probable	10	3	Recovered
26	55/M	India	Cough, dyspnea, AMS	ELISA, IFA	Possible	N/A	N/A	Recovered
26	35/M	India	Cough, dyspnea, AMS	ELISA	Possible	7	N/A	Death
27	76/M	China	Dysuria, fatigue, anorexia, chest tightness, cough	mNGS	Probable	0	5	Recovered
28	75/M	India	AMS, hemiparesis, cough, SOB	ELISA	Possible	7	N/A	Recovered
29	52/F	India	Vomiting, headache, leg pain, myalgia, dysuria	Serology	Possible	7	6	Recovered
PC	65/F	Japan	Fatigue, dry mouth	IFA	Probable	15	None	Recovered

AMS = altered mental status; F = female; Hd = hospital delay; IFA = indirect immunofluorescence assay; M = male; Metacap = metagenomic capture sequencing; mNGS = metagenomic next-generation sequencing; PC = present case; PCR = polymerase chain reaction; Pd = patient delay; RFA = rapid fluorescent antibody assay; SOB = shortness of breath. Diagnostic accuracy was classified as probable (PCR, Metacap, or mNGS, or serological analysis revealing a ≥4-fold rise in paired antibody titers or high acute-phase titers with serotype identification), or possible (serological positivity without detailed methodological or titer information).

To assess diagnostic accuracy in atypical cases of scrub typhus, diagnostic approaches were classified into two categories: probable and possible. Probable diagnosis was based on DNA-based methods, including polymerase chain reaction, metagenomic capture sequencing, or metagenomic next-generation sequencing, and otherwise relied on serological evidence with significant antibody titer elevation (≥4-fold increase in paired samples or a single acute-phase titer ≥320).^30^ A possible diagnosis was defined as only serological positivity by immunofluorescence assay, enzyme-linked immunosorbent assay, or rapid fluorescent antibody testing without detailed methodological or titer information. In the present review, the majority of cases (62%; 18/29) were classified as possible, whereas a smaller proportion (38%; 11/29) met the criteria for a probable diagnosis, underscoring the substantial reliance on serological testing in clinical practice. Although serology is widely accessible, it is susceptible to false positive results due to cross-reactivity and false negative results due to limited sensitivity, even when epidemiological exposure and clinical suspicion are strong. Although molecular methods provide higher diagnostic specificity, their sensitivity may be affected by specimen type and timing of sampling. These findings underscore the limitations of current diagnostic approaches and emphasize the importance of an integrated strategy that combines epidemiological context with both serological and molecular testing, particularly in atypical presentations lacking classic clinical features.

Of note, elevated serological titers against the Hirano/Kuroki strains suggest *Leptotrombidium scutellare*–mediated scrub typhus, consistent with the patient’s exposure history in an endemic area of Kyushu, Japan. The 4-fold rise in the *O. tsutsugamushi* IgM immunofluorescence assay titer against the Karp strain (Table 1), which is associated with *Leptotrombidium pallidum*–mediated scrub typhus endemic to northeast Japan, was considered to represent a cross-reaction.

As summarized in Table 2, treatment initiation in atypical scrub typhus was often delayed (5–15 days), reflecting diagnostic uncertainty at both the patient and healthcare levels. Fatal outcomes were reported in 17% (5/29) of cases, including young and otherwise healthy adults,^16^^,^^20^^,^^26^ whereas spontaneous resolution without antibiotic therapy was also observed,^11^ including the present case. These contrasting outcomes highlight the heterogeneous clinical course of scrub typhus and underscore the importance of timely recognition and management.

The present case highlights the diagnostic challenges of scrub typhus with atypical manifestations and underscores the central role of clinical judgment informed by epidemiological context, including geographic and exposure history. In endemic areas, clinicians should maintain a high index of suspicion even in the absence of classic findings such as fever, rash, or eschar. When available, a multimodal diagnostic approach incorporating both serological and molecular methods should be pursued. Given the potential for severe outcomes, timely and appropriate treatment, including preemptive therapy when clinically warranted, should not be delayed in endemic settings when clinical suspicion is sufficiently high.
